# Preference Ranking Procedure: Method Validation with Dogs

**DOI:** 10.3390/ani10040710

**Published:** 2020-04-19

**Authors:** Han Li, Rachel Wyant, Greg Aldrich, Kadri Koppel

**Affiliations:** 1 Center of Sensory Analysis and Consumer Behavior, Department of Food Nutrition Dietetics and Health, Kansas State University, Manhattan, KS 66506, USA; hanl@ksu.edu; 2 Department of Animal Science and Industry, Kansas State University, Manhattan, KS 66506, USA; rwyant@merrickpetcare.com; 3 Department of Grain Science and Industry, Kansas State University, Manhattan, KS 66506, USA; aldrich4@ksu.edu

**Keywords:** palatability, dog food, ranking, preference, validation

## Abstract

**Simple Summary:**

In order to provide more test options to determine the preference of dogs regarding pet food products, a preference ranking procedure was proposed previously and then validated in this study. The validation of the procedure was conducted by repeating the main portion of the preliminary test with a one-year gap and the results from both studies were compared. The results showed a general agreement and proved that the preference ranking procedure is capable of showing consistent results.

**Abstract:**

The growth of the number of pet products and the pet food industry is continuous. This is partially driven by palatability and perceptions of preference. A preference ranking procedure for dogs has been proposed in order to suggest a more efficient method to study the palatability of food products for dogs. This method was developed based on the assumption that (1) dogs would be more motivated to solve a puzzle for foods that they preferred and (2) the order in which the dogs obtained the treats from the puzzles would indicate the ranking of their preferences. This current study included a validation test that was conducted with 12 dogs to determine if the proposed method was reliable. The validation followed the same procedure as the preliminary test for most parts and dedicated a separate phase each for training, fats, proteins, starches, and complex diets. The results from phases 2 to 4 showed a similar pattern with the preliminary test: Fish oil was preferred over lard, liver over beef, and corn over chickpea. The results from phase 5 showed that the ranking of the combination of the ingredients reflected the preference of the dogs for individual ingredients. As a result, this method was concluded to be reliable.

## 1. Introduction

The pet industry has been growing steadily due to the high number of households that own companion animals. In the US, two-thirds of the households own at least one companion animal and dogs are one of the most popular choices [1]. Dog owners spend more on pet food compared to all other pet-related costs [1]. Palatability of the food has a prominent impact on their purchasing intensions [2]. 

To fulfil the pet owners’ desire to see their pets consume their food with gusto and vigor, manufacturers often evaluate palatability of the food products to guide product development. The most common methods are single-bowl and two-bowl method techniques. The single-bowl method measures the amount of food consumed by the subjects/pets and determines whether the food is acceptable [3,4]. The two-bowl method is a forced choice method, which compares which food was consumed in the greater quantity when two food choices are offered simultaneously [4,5]. However, both tests have limitations, such as the presence of forced choice, which can possibly alter the true result [4], or the limited number of samples being evaluated. Since companion animals are non-verbal, gaining an understanding regarding their preferences or liking for a particular ingredient or additive must be determined through behavioral observation. To do so in a quantitative manner requires separation of individual components within a range of food options. This inherently relies upon offering more than one or two choices simultaneously. In order to develop a new method that uses a pet’s motivation for preferred flavors or to explore the dogs’ preferences, the Preference Ranking Procedure for dogs was proposed and a preliminary test was conducted [6]. This novel method suggests that more than two samples can be presented to the dog simultaneously and, based on the sample selection order, a ranking can be generated. This ranking is translated to indicate the order of flavor and aroma preference of the dog. The results from the preliminary test showed that this method could be used to provide information regarding ingredient preferences of dogs. However, the method has not been validated thus far.

Validation is necessary for many development processes and it can be conducted in different formats. Normally, the validation study is conducted by comparing the results collected from different resources or conditions as the initial study, such as the method in Reference [7], data analysis in Reference [8], subjects in Reference [9], and the time, etc. The purpose of a validation test is to determine the performance of the developed subject (method, equipment, theory, etc.) in accordance to its intended use [10]. In order to explore the reliability of the preference ranking procedure, a validation test was needed. 

Therefore, the objectives of this study were to duplicate the preliminary test and determine whether the preference ranking procedure is reliable and consistent.

## 2. Materials and Methods 

The subjects and test procedures were the same as described in the preliminary test [6] and are described along with modifications below. This research was approved by the Institutional Animal Care and Use Committee at Kansas State University (IACUC #3722). 

### 2.1. Subjects 

Twelve young adult (2 to 4 years old) beagle dogs (4 females and 8 males) with an average weight of 11 kg ± 1.2 kg that were used in the previous preliminary test [6] participated in the validation study. They were housed at Kansas State University Large Animal Research Center (Manhattan, KS, USA) under ambient environmental conditions (20 °C; 60% Relative humidity). Dogs were housed in pairs throughout the study in dog runs (7.8 square meter inside run with an attached 18 square meter outdoor run). During 25 days of study period, a standard maintenance diet in amounts to maintain body weight were fed to the dogs twice daily at 07:00 and 11:00 and removed following the 11:00. feeding. The removal of the diet was expected to increase motivation and interest of the dogs for the ranking test. The test took place from 16:30 to 18:00 daily. Each dog was led to the testing space individually to a room adjacent to their pens. The room was a noise-free and smell-free environment, which eliminated the distraction from the barking and smell of the other dogs. A testing space was partitioned with fences to form a 1.5 × 1.5 m square space in the room, which allowed the dogs to be contained and promote focus on the task.

### 2.2. Methodology

At the beginning of the test, the researchers led one of the dogs to the testing space and introduced them to the five treats contained in hollow rubber toys. The toys were composed of rigid rubber in an abbreviated cylinder with a hole through the length of the interior where the treat can be hidden (kong; KongTM, Golden, CO, USA). The dog was directed to sniff the rubber toys that contained the different treats by having the rubber toys held in front of their nose (approximately 2 s per toy). After all 5 toys were sniffed by the dog, one researcher led the dog to the start-point located in the opposite corner of the testing space, approximately 2 m away from the toys. The other researcher placed the toys in a row on the floor approximately 10 cm apart from each other. The order of the treats being set up on the floor and the assigned number of the toy for each treat were completely randomized. 

One researcher stayed within the fence with the dog to remove the empty toy after each treat had been extracted by the dog during the test and the other researcher remained outside the fence to record the time and order of the consumption of each treat. The timer was started when the dog was released to approach and extract the treats from the puzzle-toys. The order of the treats being chosen (extracted and consumed) by the dog was considered the ranking order of preference (1 through 5). For example, the first treat consumed by the dog was scored a 1 and considered most preferred and the treat consumed last by the dog was scored a 5 and considered least preferred. If the last treat was not consumed, it was scored a 5 and considered as least preferred. In the event that more than one treat was ignored, the results were recorded as incomplete and were not included in the data. After the dog finished the test and was returned to their pen, another dog was led to the room to start their test. The order of the dogs led to the testing space remained the same to eliminate external distractions, such as unfamiliar odors. The testing space was cleaned between dogs, if necessary. The test was repeated for 5 sequential days for each phase to allow dogs to associate aroma with flavor and confirm the rank order. Each of the 60 toys were labeled sequentially and distinctly with numbers 1 to 60. After each day of testing, the rubber toys were washed with hot water and soap twice and air-dried overnight prior to use to eliminate odor and other residue (hair, saliva, etc.). 

When the dog was not interested in the task, researchers encouraged/guided the dog in order to continuously train them. When guiding was needed, the data was not included in analysis due to potential existence of bias during the test. Within 30 s from releasing the dog, if the dog was not interested in the task, researchers tapped the toy on the floor to create noise and attract the dog. If after 1 min from releasing the dog they were not on task, researchers removed the treat from the toy and allowed the dog to sniff the treat in order to attract them with the aroma. Then the treat was placed back in the toy once the dog showed interest and the test continued. If the dog was not able to start/continue the task 2 min after being released or having extracted the treat, the test was ended.

Five phases were included in this validation test. For each phase, the same test with the same treats was repeated for 5 days as five replications. The first four phases were consistent with the preliminary test [6]. For the first 5 days of the study (phase 1), which was the training/practicing phase, the dogs were introduced to the test procedure using five commercial dog treat products, followed by phases 2, 3, and 4 of evaluation of different fats, starches, and proteins, respectively, and a final phase to evaluate complex diets with combinations of ingredients tested in previous phases. 

**Figure 1:**
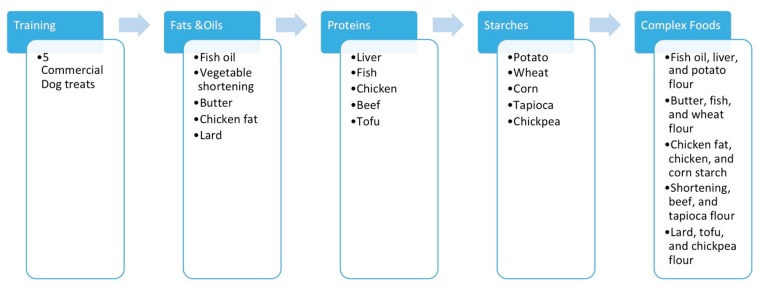
Visualization of the test flow in phases 1 (Training) to 5 (Complex Foods).

During the first phase, three commercial dog treat products were used for training/practice. On the last day of this phase, a performance evaluation was conducted in order to monitor the ability of the dogs to discern differences within the limits of the study protocol. The test consisted of six segments, including if the dog needed assistance to sniff toys prior to the test, if the dog sniffed toys while choosing the treats, if the dogs needed guidance from researchers during the study (attract dogs to kongs with noise, letting the dog sniff the treat), and if the dog showed interest towards toys and treats. Each dog was scored for the behavior mentioned in each category. Each category contributed 1 point, depending on the nature of the behavior. When the dog successfully completed more than half of the tests, their ability of performing the test was considered acceptable. No preference data was collected in this phase, since its purpose was simply to introduce the procedure to the dogs and work out timing for the procedure. However, qualitative observations, such as the technique to extract the treat and urinating behavior, were noted to provide insights on the subjects’ progress in adapting to the procedure. For phases 2 to 5, five different treats were ranked by the dogs, respectively. In phases 2 to 4, the dogs completed the ingredient evaluations, while phase 5 was a final test with a complex diet (Figure 1).

### 2.3. Samples

Generic dog treats (CON; Great Choice, Phoenix, AZ, USA) and two premium baked treats (PRE; Old Mother Hubbard Baking Co., Tewksbury, MA, USA) were used in phase 1 (training). Three of the toys were filled with a random selection from the CON biscuits, which contained three different colored biscuits, and the other two were filled with a biscuit from each of the PRE treats. 

In phases 2 to 5, the treats used in each phase were made the day prior to the first day of each phase in Center of Sensory Analysis and Consumer Behavior at Kansas State University for the each of the 5 days of evaluation (Table 1).

The treats were made by first combining the dry ingredients and then cutting the shortening/fat into the dry ingredients. Then the liquid ingredients were mixed together and slowly added into the dry ingredients. The dough was then mixed by hand until the ingredients were combined and the dough was formed. The dough was rolled into a 10 mm thick sheet and cut into 15 × 20 mm rectangles. The size was adjusted from Reference [6] because it was difficult for the dogs to extract the larger treat. The treats with smaller size were able to be extracted freely. The treats were then placed on a baking sheet with parchment paper and baked in a convection oven at 175 °C for 15 min. The treats were stored at room temperature (20 °C; 60% RH) in gallon-sized plastic bags (Ziploc, S.C. Johnson, Racine, WI, USA) once cooled and were stored until use in the evaluation. 

For each of the phases 2 to 4, within each recipe, one ingredient was exchanged with different options within the ingredient category (Table 1). In phase 2, fats and oils were the selected categories of ingredients to be evaluated: fish oil (Omega Protein, Houston, TX, USA), chicken fat (IDF Inc., Springfield, MO, USA), lard (Armour, Phoenix, AZ, USA), butter (Land O’Lakes, Minneapolis, MN, USA), and vegetable shortening (Crisco, Orrville, OH, USA). In phase 3, different protein sources were evaluated. These included beef (70:30 ground beef, Dillons, Hutchinson, KS, USA), chicken (Dillons, Hutchinson, KS, USA), fish (Salmon, Dillons, Hutchinson, KS, USA), tofu (Dillons, Hutchinson, KS, USA), and chicken liver (Tyson, Springdale, AZ, USA). Starch and grain ingredients were evaluated in phase 4, including whole wheat flour (Gold Medal, Minneapolis, MN, USA), chickpea flour (Garbanzo Bean Flour, Bob’s Red Mill, Milwaukie, OR, USA), potato flour (Bob’s Red Mill, Milwaukie, OR, USA), corn starch (Argo, Cordova, TN, USA), and tapioca flour (Bob’s Red Mill, Milwaukie, OR, USA). 

In phase 5, treats were made with ingredients from the previous three phases in accordance to how they were ranked. Specifically, the most preferred ingredients within each category were added to the basal recipe and identified as treatment 1. The second most preferred ingredients were combined as treatment 2; the third, fourth, and fifth preferred were used to produce treatments 3, 4, and 5, respectively. The five resulting combinations were: (1) Fish oil, liver, and potato flour; (2) butter, fish, and wheat flour; (3) chicken fat, chicken, and corn starch; (4) shortening, beef, and tapioca flour; (5) lard, tofu, and chickpea flour. 

### 2.4. Data Analysis 

The ranked data from each of these phases was analyzed by a Friedman analysis of variance [11] using the XLStat version 2019.3.2.61545 (Addinsoft, New York, NY, USA). The time differences of each phase were analyzed by one-way Analysis of Variance (ANOVA) with SAS® statistical software (SAS® version 9.3, SAS Institute Inc., Cary, NC, USA) using Procedure for Generalized Linear Mixed Models (PROC GLIMMIX) to determine significant differences among samples. The statistical significance of differences was defined as *p* ≤ 0.05.

In order to validate the data from this study, additional comparative analysis was conducted. The data collected in the current study was compared to the data collected in the preliminary preference ranking procedure [6]. This analysis included a comparison of two samples for phases 2, 3, and 4 by comparing individual fat, protein, and starch ranking scores. For example, fish oil ranking scores from the preliminary study were compared to the fish oil ranking scores from the validation study. This was done using the Wilcoxon test and XLStat version 2019.3.2.61545 (Addinsoft, New York, NY, USA). The statistical significance of differences was defined as *p* ≤ 0.05.

## 3. Results

### 3.1. Phase 1

Compared to the preliminary test [6], some main differences regarding the training process were observed. First, the dogs discovered the proper techniques to extract the treat out of the puzzle within the first 2 days of the training phase. The common techniques used included: (1) bouncing the puzzle by picking it up and dropping it on the floor; (2) rolling the puzzle by pushing it; and (3) reaching inside puzzle with tongue movement. In most cases, combinations of the three techniques were efficiently used. On the last day of the training phase, the dogs who completed the task finished the task within 3 min (Table 2). Second, unlike during the preliminary test, most dogs were able to start the test without guidance or attracting them to the puzzles. Thirdly, for the validation test, the dogs did not spend as much time sniffing and exploring the testing room as in the preliminary test. 

### 3.2. Phases 2–5 and Validation Analysis

The preference ranking during phase 2 using fats and oils indicated a preference (*p* < 0.05) for the fish oil relative to the shortening and lard treatment (Table 3). Butter was preferred over lard, but not over chicken fat and shortening. The chicken fat was not found significantly different from the other samples (*p* > 0.05). When the data from each day was analyzed separately, no significant preference was observed within duration of the phase.

In phase 3 of the study, dogs showed a preference (*p* < 0.05) for the chicken liver relative to the all four other treatments and fish; chicken and beef were preferred over tofu (Table 3). For each individual day, only day 2 showed that the sample prepared with chicken liver was significantly preferred over the chicken and tofu samples.

The results of phase 4 showed that the dogs preferred (*p* < 0.05) the potato flour treatment in comparison to the wheat flour, corn starch, tapioca flour, and chickpea flour treatments (Table 3). The wheat flour and corn flour treatments were preferred over chickpea flour. On day 5, the dogs significantly preferred the sample prepared with potato flour over the samples with tapioca flour and chickpea flour. The preference ranking was not significant for the other four individual days.

In phase 5, the result indicated that sample 1 was significantly preferred over samples 2 to 5, while sample 2 was preferred over samples 4 and 5 and sample 3 was preferred over sample 5 (*p* < 0.0001; Table 3). For each individual day, significant differences on the preferences were observed on day 2, day 3, and day 5. On day 2, sample 1 was only preferred over sample 4, while, on day 3, sample 1 was preferred over both sample 4 and 5. Sample 1 was preferred over sample 2, 4, and 5 on day 5.

The validation analysis showed that there were no significant differences in the ranking scores of the individual samples tested between the preliminary study [6] and the current study in phases 2 and 3 (*p* < 0.05). Further, wheat, corn, tapioca, and chickpea scores were found to be not significantly different between the two studies (*p* < 0.05). The only sample scores that were found to be significantly different between the two studies was potato.

### 3.3. Length of Tests

**Figure 2:**
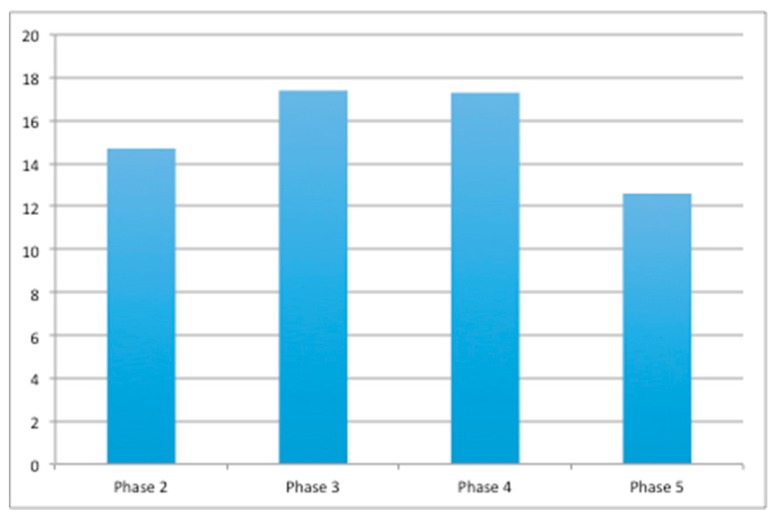
Average time (s) the dogs spent on each toy from phase 2 to 5.

After the training phase, the total elapsed time for each dog to complete the test was less than 3 min. The average length of phase 5 was significantly shorter than phases 3 and 4 (*p* = 0.0003, Figure 2). 

**Figure 3:**
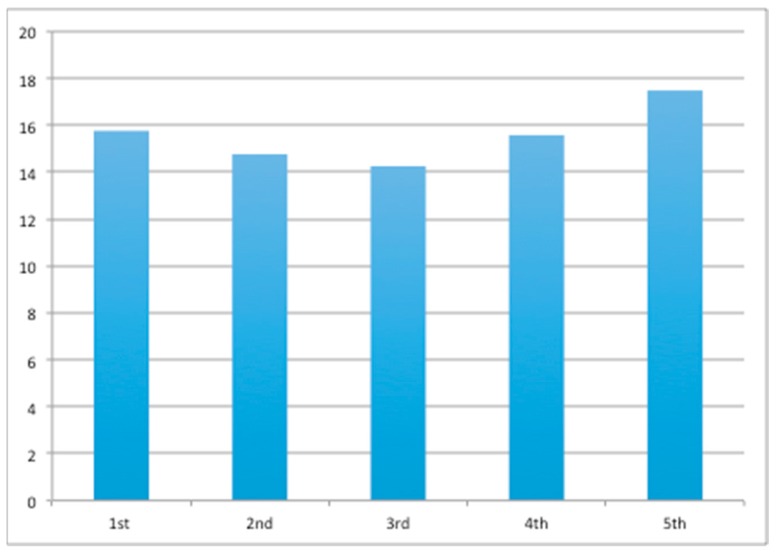
Average time (s) the dogs spent on each toy over testing in phases 2–5.

The time the dogs spent between consuming treats were relatively stable (*p* = 0.1030, Figure 3). There was a slight decrease in time between the sample from the first toy to the third toy and a slight increase from the third toy to the fifth toy. 

## 4. Discussion

Based on the comparison of behavioral observations between the preliminary test and the validation test, the dogs showed higher efficiency in completion of the validation study. Since the dogs were the same animals used in the preliminary test, it is possible that the memory from the procedure from before was maintained during the 12-month period between tests and suggests that this method may be capable of long-term use. If this method is continuously used on a regular basis, training periods may not be necessary, since the dogs would gain the ability of extracting treats/food from the puzzle from the initial training process. As a result, if the relatively long training period was a concern for time and technician investment using this method, an extensive re-training session may not be necessary. Based on the experience from the preliminary test, the 1.5 × 1.5 m test space formed at the beginning of the study may also contribute to the reduction of time during the training process.

There were some similar patterns of behavior, as in the preliminary test with dogs who failed to complete the test. For example, these dogs ignored the puzzles/treats, interacted with researchers, and sniffed unrelated objects. Most of the dogs who qualified in the preliminary test also showed acceptable performance in this validation test, with one exception. The possible reason for the one dog to perform differently in the two tests may potentially be associated with the change of living environment from being housed individually to being housed in pairs, which can potentially result in stress.

For phase 2, overall, the results supported the preliminary test results and showed more significant differences, since the preliminary test only determined that the fish oil treatment was preferred over lard. This result supported the theory that butter and fish oil aroma potentially attracted the dogs [12]. As was observed in the preliminary test, the chicken fat and lard were not preferred relative to the vegetable shortening.

For phase 3, some differences associated with dogs’ preference of protein source were observed between the preliminary test and the validation test. In the preliminary test, chicken, fish, and tofu were preferred similarly, which was unexpected. The result from the validation test showed that animal-based protein was preferred over plant-based protein. This agrees with the research of Houpt and Smith [13]. However, since this result was slightly different from the preliminary test, more replications or tests with other plant-based protein sources should be considered in order to determine if animal-based protein sources would actually be generally preferred over plant-based protein sources. Additionally, the quality of the ingredients and production lots can also potentially affect the results.

The preference ranking result for phase 4 of the validation test was different from the preliminary test due to the switch of the position between potato flour and corn flour. Even if grain-added products have more overall aromatics than grain-free products [14], the different age of the raw ingredients in the potato flour sample may have changed the intensity of overall aromatics. However, no existing research was found that would explain if the dogs were attracted to overall aromatics intensity or a specific aroma note in dog food. To further examine the result, additional research should be conducted.

For phase 5, there was no existing data to compare against. Still, the result supported the assumption that the dogs were capable of differentiating the ingredients and expressing their preferences on the types of ingredients as a combination in a similar fashion to the individual ingredients. 

The time reduction trend from phase to phase was not as obvious as in the preliminary test. One explanation for why phase 5 of the preliminary test was so short could be that the size of the commercial food samples was smaller and the shape was different from the baked treats used in phases 2 to 4. This could result from the extracting process being easier and smoother. In the validation test, the dogs did not spend much time exploring the testing environment. This may have indicated their familiarity with the testing space. The short testing time in phase 5 generally agreed with the preliminary test, except that for phase 2 a shorter adjustment period was needed due to the previous test. However, the differences in the average times across phases was less than 5 s; thus, it is reasonable to conclude that once the training was completed, the speed of completing the tests was relatively stable and consistent.

Obtaining and consuming the first toy/treat after being released by the researcher did not take less time than other treats when compared to the preliminary test, and the time spent on each toy was more stable than during the preliminary test. This could be the result of the increased dog confidence and relaxation levels, and this may have decreased the excitement and stress, leading to more focus to complete the test. The dogs were using their own pace to finish the study, which possibly helped the decision-making process. For the validation test, the differences in preferences were generally more significant when comparing the preliminary to the validation test, and this may be a result of a more relaxed decision-making process.

The composition of the treat could also be a factor that influences the dogs’ choices. It is possible that the impact of one category of ingredients (fat/protein/starch) was more substantial on the dogs liking than the other categories. When comparing a high-fat diet to a high-carbohydrate diet, the high-fat diet resulted in higher voluntary dry matter intake and higher calorie intake than the high-carbohydrate diet [15]. Further application of the preference ranking method with dogs should be conducted to determine what the impact of the ratios of different ingredients might be. Additionally, a sensory descriptive study with the dog food lexicon published by Di Donfrancesco et al. [16] should be conducted in order to determine how the ratios of the ingredients might affect the individual aromatics of the dog food and the overall intensity of the aromatics. With proper statistical analysis, the results from the preference ranking procedure and the descriptive analysis can potentially determine what aromatic notes tend to drive dog liking. Further, additional studies need to be conducted using dogs who live in pet owners’ homes in order to determine whether the preference ranking procedure can be used to make conclusions beyond the controlled laboratory and upon ingredient composition alone.

One limitation of the preference ranking procedure would be that this method is relatively labor-intensive when compared to the one-pan and two-pan methods, which do not necessarily require constant presence of researchers during the tests. In the ranking test, two researchers were required to operate and manage the entire study. During the testing process, one researcher was needed to stay outside of the testing space to record the order and time of the treats being extracted and consumed. The other researcher had to stay in the testing space to remove the puzzle after the treats being extracted to avoid the dogs “revisiting” the empty puzzles. However, the observations from the researchers can potentially provide more insights on the behaviors of the dogs and ensure the accuracy of the tests. Another limitation would be the forced-choice effect, which is still present even if the treats are not the dogs’ main meal of the day and the dogs are given a choice not to consume any treats. Only completed test results were collected for data analysis, and the nature of a ranking test is still a forced-choice test. 

From the results of the preliminary test and the validation test, the preference ranking procedure method is reliable and potentially suitable for continuous use for different studies. The dogs were able to be re-trained to maintain the speed and techniques after a 12-month break period from the initial testing, which proved this method to be extendable. The results from the validation test in general were similar to the results from the preliminary test and showed even more differences among samples, thereby proving this method to be reliable. Preference ranking studies, such as this one. also have the advantage of being highly efficient, as more samples can be evaluated at the same time. 

## 5. Conclusions

A validation test for the preference ranking procedure was conducted after the completion of the preliminary test with five phases, including training, evaluation with three different categories of ingredients, and evaluation of food with combinations of preferred/not preferred ingredients. The training and evaluation with three different categories of ingredients phases repeated the same procedure and materials as the preliminary test. The preliminary test showed that this method could compare dogs’ preference to five samples simultaneously and provide a general conclusion on which ingredient was preferred or liked over the other. This validation test, which was a semi-duplication, provided evidence that this method is reliable because, overall, it led to similar results with the preliminary test and the validation even provided more clear-cut results. 

## Figures and Tables

**Table 1:** Ingredient % composition for treats evaluated by dogs in phases 2–5.

Ingredient	Phase 2	Phase 3	Phase 4	Phase 5
Whole wheat Flour (Starch source replacement)	10.08	24.33	28.82 ^c^	25.84 ^c^
White (all purpose) flour	40.03	24.33	2.88	25.84
Corn meal	12.59	12.16	0.00	0.00
Salt	0.50	0.49	0.58	0.52
Baking soda	0.25	0.24	0.29	0.26
Dry milk	1.51	1.46	1.73	1.55
Sodium bisulfite	0.002	0.002	0.002	0.002
Dry yeast	0.002	0.002	0.002	0.002
Shortening (Fat source replacement)	7.56 ^a^	7.30	8.65	7.75 ^a^
Molasses	4.03	3.89	4.61	4.13
Water	23.10	16.06	26.51	23.77
Meat (protein source replacement)	0.00	9.73 ^b^	0.00	10.34 ^b^

^a^ Fat replacements included vegetable shortening, fish oil, chicken fat, butter, or lard; ^b^ Proteins included fish, beef, chicken, liver, or tofu; ^c^ Starches included whole wheat flour, tapioca flour, potato flour, corn starch, or chickpea flour.

**Table 2:** The performance evaluation result of the dogs at the end of phase 1.

DOG	Need Assistance to Sniff Toys Prior to Test (Yes = 0, No = 1)	Sniff Toys while Choosing (Yes = 1, No = 0)	Need Guidance towards Toys (Yes = 0, No = 1)	Need to Sniff Treats Directly (Yes = 0, No = 1)	Interest in Toys (Yes = 1, No = 0)	Interest in Treats (Yes = 1, No = 0)	Total	Length of Study (s)
F1T	1	1	1	1	1	1	6	90.0
F1P	1	1	1	1	1	1	6	71.0
F2C	1	1	0	0	1	1	4	175.0
F2L	0	0	0	0	0	0	0	n/a *
M4W	0	0	0	0	0	0	0	n/a
M8C	0	1	1	1	1	1	5	98.0
M5P	1	1	1	1	1	1	6	84.0
M5T	0	0	0	0	0	0	0	n/a
M6J	0	1	0	1	1	1	4	180.0
M6P	0	0	1	1	1	1	4	140.0
M7D	0	0	0	0	0	0	0	n/a
M7F	0	0	0	0	0	0	0	n/a

* n/a—Subject did not complete test.

**Table 3:** The effect of different treatments (fat, protein, starch, and complex food) on rank order preference in dogs in phases 2–5.

Phase	Treatments				
2: Fats and Oils	Fish Oil	Butter	Chicken Fat	Vegetable Shortening	Lard
	2.18 ^a*^	2.72 ^ab^	3.10 ^abc^	3.32 ^bc^	3.68 ^c^
3: Proteins	Liver	Fish	Chicken	Beef	Tofu
	1.64 ^a^	2.57 ^b^	3.12 ^b^	3.35 ^b^	4.32 ^c^
4: Starches	Potato	Wheat	Corn	Tapioca	Chickpea
	1.44 ^a^	2.79 ^b^	3.04 ^b^	3.47 ^bc^	4.27 ^c^
5: Complex Food **	Sample 1	Sample 2	Sample 3	Sample 4	Sample 5
	1.35 ^a^	2.78 ^b^	2.80 ^bc^	3.66 ^cd^	4.41 ^d^

* within a row, sample average ranking scores with a different letter were significantly different (*p* < 0.05); ** Sample 1: fish oil, liver, potato flour; Sample 2: butter, fish, wheat flour; Sample 3: chicken fat, chicken, corn starch; Sample 4: shortening, beef, tapioca flour; Sample 5: lard, tofu, chickpea flour.
